# The Interplay of Phonons and Subgap States Governs Ultrafast Carrier Dynamics in Layered HgPSe_3_

**DOI:** 10.1021/acs.jpcc.6c03448

**Published:** 2026-10-01

**Authors:** Alessandro Baserga, Miłosz Rybak, Micol Bertolotti, Antonella Treglia, Samim Sardar, Valentino Jadriško, Matteo Ghezzi, Giuseppe Maria Paternò, Cosimo D’Andrea, Annamaria Petrozza, Zdenek Sofer, Artur P. Herman, Robert Kudrawiec, Giulio Cerullo, Giulia Folpini, Nikolas Antonatos, Christoph Gadermaier, Stefano Dal Conte

**Affiliations:** † Department of Physics, 18981 Politecnico di Milano, 20133 Milan, Italy; ‡ Department of Semiconductor Materials Engineering, 49567 Wrocław University of Science and Technology, Wybrzeże Wyspiańskiego 27, 50-370 Wrocław, Poland; § Istituto Italiano di Tecnologia, Milano 20134, Italy; ∥ Department of Inorganic Chemistry, University of Chemistry and Technology Prague, Technická 5, 166 28 Prague 6, Czech Republic; & Department of Chemistry, National Institute of Technology Warangal, Telangana 506004, India

## Abstract

Two-dimensional van der Waals materials display rich nonequilibrium physics due to their reduced dimensionality and strong light–matter interactions. Layered metal phosphorus trichalcogenides (MPX_3_) feature bandgaps spanning the visible to ultraviolet range and exhibit a complex interplay between excitons, phonons, and defects. Here we use static and time-resolved photoluminescence and femtosecond broadband transient reflectivity to investigate the temperature-dependent ultrafast carrier dynamics in layered HgPSe_3_, a semiconductor with a quasi-direct bandgap near 2 eV. We identify distinct sub-bandgap photoluminescence features that become prominent at low temperature. Their temperature and fluence dependence strongly indicate radiative recombination involving localized states. The transient reflectivity map displays three features associated with interband optical transitions in the visible range, as corroborated by density functional theory band structure calculations. These features exhibit fluence-dependent decay dynamics, indicating a competition between carrier localization into sub-bandgap states, state-filling effects that limit the available electronic phase space for hot-carrier relaxation, and phonon-bottleneck-limited carrier cooling. Our results provide new insights into the ultrafast carrier dynamics of HgPSe_3_ and establish a framework for understanding the role of sub-bandgap states in MPX_3_ semiconductors, with potential implications for broadband optoelectronic and photodetection applications.

## Graphical abstract

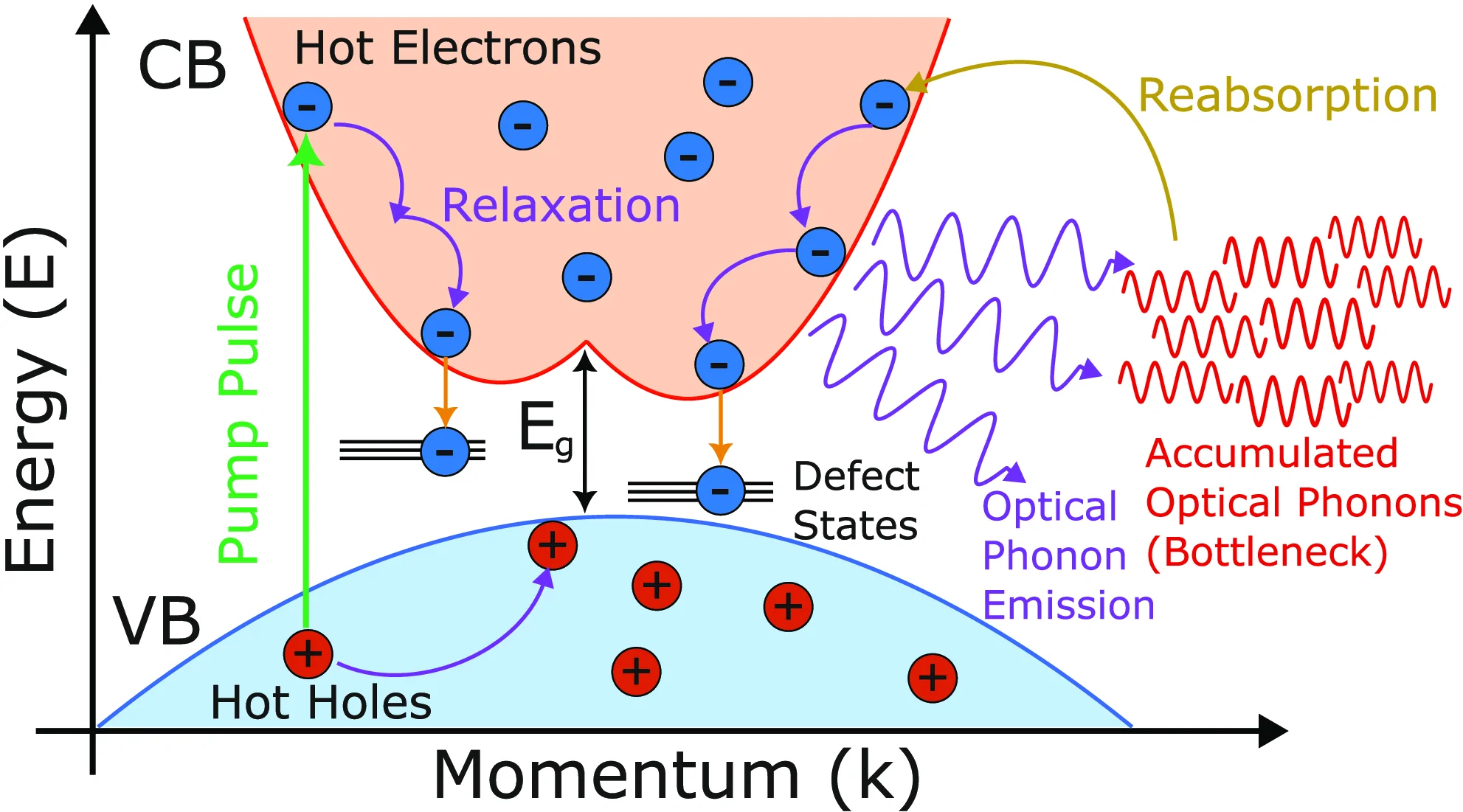

## Introduction

Among the broad family of layered van der Waals (vdW) materials, transition metal phosphorus trichalcogenides (MPX_3_, where M = transition metal and X = chalcogen) have gained increasing attention. These materials, which crystallize in a layered monoclinic or rhombohedral structure,^1,2^ show a peculiar interplay between their structural, electronic, magnetic, and optical properties.^2,3^ These compounds exhibit a wide range of behaviors depending on the choice of metal and chalcogen elements, including long-range magnetic ordering, Mott insulating behavior, and large optical nonlinearities.^4–7^ In addition, their optical bandgaps span a broad spectral range from approximately 1.3 to 3.5 eV,^8,9^ covering the visible to near-ultraviolet spectrum and enabling potential integration into photodetectors, solar cells, and spintronic devices.^10–14^ This versatility makes MPX_3_ compounds a valuable platform for exploring fundamental light-matter interactions and for engineering next-generation optoelectronic devices.

HgPSe_3_, a member of the MPX_3_ family, features a van der Waals layered structure, a quasi-direct bandgap of approximately 2 eV, and strong light absorption, making it a promising candidate for broadband photodetection from the visible to the X-ray range.^11,14,15^ Recently, combined time-resolved microwave conductivity and time-resolved photocurrent measurements have revealed a surprisingly fast photoresponse in the nanosecond range that is independent of the applied electric field, underscoring its potential for high-performance, broadband photodetectors.^12^ However, device performance depends critically on carrier lifetimes, recombination pathways, and energy relaxation dynamics. Ultrafast optical spectroscopies provide direct access to these processes, disentangling the roles of excitons, defect states, and phonon scattering mechanisms. In particular, the interplay among carrier cooling, phonon-driven scattering and defects-mediated recombination critically governs charge transport efficiency and device response times.

Here, we investigate the static and ultrafast optical response of HgPSe_3_ through the combination of stationary and time-resolved optical spectroscopies. Excitation fluence- and temperature-dependent photoluminescence (PL) measurements show that the highest-energy emission peak around 2 eV originates from excitonic and free-carrier radiative recombination, while two broad sub-bandgap emission features centered at approximately 1.8 and 1.3 eV emerge at low temperatures. Although the temperature and fluence dependence of these features is consistent with radiative recombination involving localized states, their microscopic origin cannot be unambiguously established based on the present experiments alone. Time-resolved PL (TRPL) and femtosecond transient reflectivity (TR) measurements provide direct insight into the nonequilibrium carrier dynamics and relaxation processes. TRPL probes the dynamics of both the band-edge and sub-bandgap emission, while TR provides complementary information on the initial carrier relaxation and higher-energy interband optical transitions. Density functional theory (DFT) calculations of the electronic band structure further corroborate the assignment of the observed interband optical transitions and provide a microscopic framework for interpreting the transient reflectivity response. Together, these results reveal the interplay between carrier localization and phonon-assisted relaxation in the out-of-equilibrium dynamics of HgPSe_3_.

## Experimental and Theoretical Methods

### Synthesis of HgPSe_3_

The synthesis procedure followed that reported previously.^11^ A stoichiometric mixture weighing 15 g of mercury, red phosphorus, and selenium (all 99.999%, Strem, Germany) was inserted into a quartz ampule with 0.25 g of HgI_2_ as a transport agent. To avoid mercury evaporation, the ampule was cooled with liquid nitrogen and sealed under a vacuum of 1 × 10^–3^ Pa. The growth process involved multiple heating stages. The ampule was first placed in a crucible furnace for 48 h, heated to 400 °C, while the cold end was kept below 200 °C. It was then transferred to a horizontal two-zone furnace. For the first 24 h, the reaction mixture was held at 350 °C with the growth zone set to 450 °C. Afterward, the charge zone was maintained at 400 °C, while the growth zone was gradually cooled from 350 to 300 °C over a period of 5 days. Finally, the ampule was opened inside an argon-filled glovebox to collect the obtained crystals.

### Static Photoluminescence

For the static PL measurements, we employed an amplified ytterbium:KGW laser from Light Conversion Ltd. (PHAROS) with an output power of 10 W, a fundamental wavelength at 1030 nm, a pulse duration of approximately 280 fs and a repetition rate of 500 kHz. The laser source was used to generate light at 515 nm by frequency doubling in a 3 mm thick β-barium borate (BBO) crystal. The pump was focused using a lens to a spot with an approximate diameter of 300 μm, and the incident fluence was varied by inserting a neutral density filter. The sample was housed in a cryostat, and its photoluminescence was recorded using a MAYA 2K PRO spectrometer (Ocean Optics).

### Time-Resolved Photoluminescence

For the time-resolved photoluminescence (TRPL) measurements, a tunable Ti:sapphire oscillator (Chameleon Ultra II Coherent) was used as the excitation source. In this case, the laser delivered a train of pulses with a duration of 140 fs at a central wavelength of 1020 nm and a repetition rate of 80 MHz. The excitation wavelength of 510 nm was obtained by second-harmonic generation in a β-barium borate (BBO) crystal. A homemade microscope was incorporated in the setup to achieve spatial resolution. A 4× objective (Nikon) was used to focus the excitation beam onto the sample resulting in a spot size of approximately 35 μm. A 530 nm long pass dichroic mirror was used to reflect the excitation beam (510 nm) into the objective. The emission signal, transmitted through a 550 nm long-pass filter, was collected and analyzed using a spectrograph (Acton SP2300 Princeton Instruments) coupled to a streak camera (Hamamatsu C5680) equipped with a synchro-scan voltage sweep module. Low temperature measurements were performed using a liquid nitrogen-cryostat (Oxford Instruments) operated under vacuum conditions. The fluorescence intensity was recorded as a function of both wavelength and time, with spectral and temporal resolutions of ∼1 nm and ∼2 ps for the 150 ps time window and ∼ 20 ps for the 2 ns time window, respectively.

### Transient Reflectivity

An amplified femtosecond Ti:sapphire laser system (LIBRA by Coherent) with a fundamental wavelength of 800 nm, pulse duration of approximately 100 fs and a repetition rate of 2 kHz was used to pump an optical parametric amplifier (OPA) for the pump pulses and to generate a white light continuum (WLC) for the probe pulses. For the pump, a visible OPA pumped by the second harmonic of Ti:sapphire generated in a 2 mm-thick β-barium borate (BBO) crystal and seeded by a WLC generated in a 3 mm-thick sapphire crystal was used, resulting in pulses centered at 510 nm with an approximate bandwidth of 10 nm. For the probe, the laser fundamental was focused into a 3 mm-thick sapphire crystal, resulting in a broadband WLC pulse with a spectrum ranging from 470 to 780 nm. The differential reflection signal, defined as $\frac{\Delta R}{R}=\frac{\left(R_{pump\;on}-R_{pump\;off}\right)}{R_{pump\;off}}$ was recorded using a fast visible camera (Stresing FLCC3001-FFT) coupled to a Princeton Instruments spectrometer as a detector.

### Computational Details

All first-principles calculations were performed using Vienna Ab Initio Simulation Package (VASP).^16^ We employed the Perdew–Burke–Ernzerhof (PBE) generalized gradient approximation functional,^17^ together with projector-augmented-wave (PAW).^18^ A plane-wave basis cutoff of 500 eV and 16 × 8 × 8 Monkhorst–Pack^19^ *k*-point grid for BZ integration were found to ensure the convergence of both the lattice constants and the electronic gaps, with a precision of 0.001 Å and 0.001 eV, respectively. A Gaussian smearing of 0.05 eV was used for BZ integrations. van der Waals interactions were included via Grimme’s D3 correction.^20^ Spin–orbit coupling (SOC) was explicitly included during the geometry optimization stage. For the evaluation of optical transitions, dipole matrix elements were computed via density functional perturbation theory (DFPT).^21^ In the excitonic calculations, we used the mBSE (model Bethe–Salpeter Equation^22,23^ ) approach within the Time-Dependent DFT (TDDFT).^24–26^ Multiple k-point grids were tested, and convergence analyses are presented in the Supporting Information. In the dielectric-dependent hybrid (DDH) scheme, the screening parameter was set to 1.26 Å^–1^, estimated from simple dimensional scaling relations of the valence electron density (the value obtained is within the range typical for semiconductors).^27^ The scissor correction to align the quasiparticle band gap was essential to properly position excitonic states relative to the band continuum.

## Results and Discussion

The PL spectrum of a bulk HgPSe_3_ crystal (its structure is reported in Figure 1A) is obtained by exciting the material with a femtosecond laser pulse at 2.4 eV and exhibits a significant temperature dependence (Figure 1B reports representative spectra at selected temperatures, while the complete temperature series is shown in Figure S2 of the Supporting Information).

**Figure 1:**
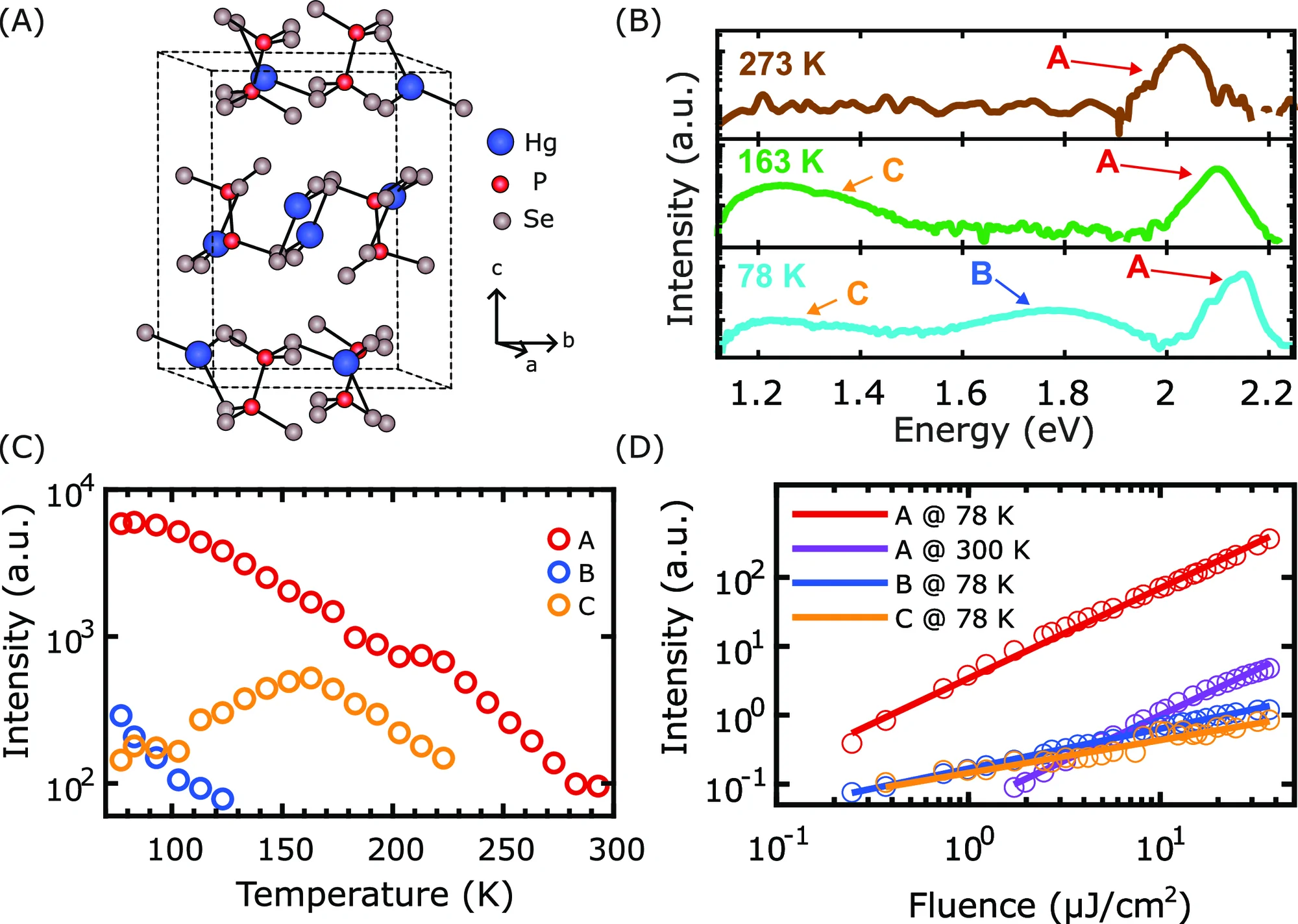
(A) Crystal structure of bulk HgPSe_3_ drawn using VESTA.^28^ (B) PL spectra at 273 K (brown), 163 K (green), and 78 K (light blue) measured at an excitation fluence of 1 μJ/cm^2^. (C) Temperature dependence of the intensity of different PL peaks. (D) Fluence dependence of the peak intensities at 78 and 300 K.

At room temperature, the PL spectrum is dominated by an emission feature at 2.01 eV, denoted as peak A (the temperature dependence of this peak is reported in Figure S1).^11^ As discussed later, this feature originates from both excitonic and quasi-direct band-to-band radiative recombination. Upon cooling, a second broad emission feature centered at 1.3 eV (peak C) emerges around 230 K, while a third peak at 1.8 eV (peak B) appears below approximately 120 K. Neither peak B nor peak C can be associated with the interband optical transitions identified by our DFT calculations or with excitonic resonances related to those transitions. Their pronounced temperature dependence, together with the excitation fluence dependence discussed below, strongly supports radiative recombination involving localized sub-bandgap states. Moreover, in MPX_3_ compounds, such localized states are commonly associated with defects i.e., phosphorus vacancies^29^ metal vacancies,^30^ and metal/chalcogen divacancies.^31^ However, the present measurements do not allow the microscopic nature of these localized states to be uniquely established. In the following, we investigate the photophysical properties of all three emission features without assuming a specific microscopic origin for peaks B and C. Figure 1C shows the temperature dependence of the integrated PL intensities. As the temperature decreases, peak A gradually blue-shifts from 2.00 to 2.15 eV because of the bandgap widening^11^ (see Figures S1, S2 and S3) and its intensity increases owing to reduced thermal dissociation and suppressed nonradiative recombination channels.^32–34^ In contrast, the intensity of peak C reaches a maximum around 170 K before gradually decreasing upon further cooling. Such nonmonotonic behavior is characteristic of radiative recombination involving localized states and reflects the competition between carrier capture, thermal redistribution, and nonradiative recombination. At low temperatures, carriers are efficiently localized in band-edge or shallow localized states, reducing the population of the emitting sub-bandgap state. As the temperature increases, thermal activation promotes carrier transfer into the emitting localized state, enhancing its population and increasing the PL intensity. Above the temperature corresponding to the maximum intensity, thermal escape from the localized state and thermally activated nonradiative recombination become increasingly efficient, leading to quenching of the emission.^35–37^ Similar temperature-dependent behavior of sub-bandgap emission has also been reported for the isostructural compound HgPS_3_.^38^

To further investigate this behavior, we extract the activation energy of the carrier escape process which quenches peak C by fitting the temperature-dependent data to the Arrhenius equation *I*(*T*) ∝ *I*_0_ exp­(*E*_a_/*k*_B_ *T*)*I*(*T*).^38^ The fit, shown in Figure S4, yields an activation energy *E*_*a*_ ∼ 70 meV. For temperatures below 170 K, the intensity of peak C decreases, while peak B becomes observable. Their temperature dependence, with decreasing intensity at higher temperatures, suggests that carrier/exciton escape from both types of defects is a thermally activated process, which is generally modeled using the Arrhenius equation. Importantly, the energetic ordering of the sub-bandgap emission peaks does not, by itself, determine their temperature dependence or, in particular, the temperature range over which the corresponding states become emissive. From the temperature dependence of peaks B and C we deduce a lower escape energy for the type of defect at the origin of peak B. We assume that also their population via exciton capture is thermally activated, as suggested for peak C by the increased intensity for higher temperature up to 170 K. An analogous mechanism can be inferred for peak B, but would need confirmation by measurements below liquid nitrogen temperature.

To further elucidate the origin of the different PL peaks, we analyze the dependence of their intensity on the incident pump fluence *F*, a commonly used approach in semiconductors.^39–42^ In particular, the emission intensity follows a power law of the form *I*(*F*) ∝ *F* ^α^ ^39^ (see Figure 1D). For the highest energy excitonic peak at *T* = 78 K, we obtain α = 1.36, which remains nearly unchanged at room temperature (i.e., α = 1.29). A power-law exponent higher than 1 is consistent with an excitonic contribution but also suggests the involvement of additional processes such as defect-assisted recombination, exciton–free-carrier interactions, and exciton–exciton interactions. This interpretation is further supported by the theoretical analysis presented in the final section of this manuscript. In contrast, the sublinear power law exponents obtained for the PL peaks B and C (α = 0.62 and α = 0.41, respectively), indicate a radiative behavior that is not proportional to the number of photogenerated carriers, consistent with emission from defect-related states.^39^

To gain further insight into the nature of the PL peaks we performed TRPL measurements. Figure 2A shows a TRPL map measured at 78 K for above bandgap photoexcitation at 2.43 eV, revealing a strong signal centered at peak A and a weaker contribution associated with peak B. We attribute the observed lack of emission around 1.3 eV to the sharp decrease in the streak camera detector sensitivity below 1.45 eV. Figure 2B shows the dynamics of peaks A and B, normalized to their maximum intensity. The empty circles represent the experimental data, while the solid lines indicate the results of a biexponential fit (see the Supporting Information for further details). Peak A forms within the response time of the streak camera (4 ps) and exhibits a biexponential decay, with both time constants increasing with increasing excitation fluence: a short one (τ_*S*_) of the order of a few picoseconds and a long one (τ_*L*_) of the order of several tens of picoseconds.

**Figure 2:**
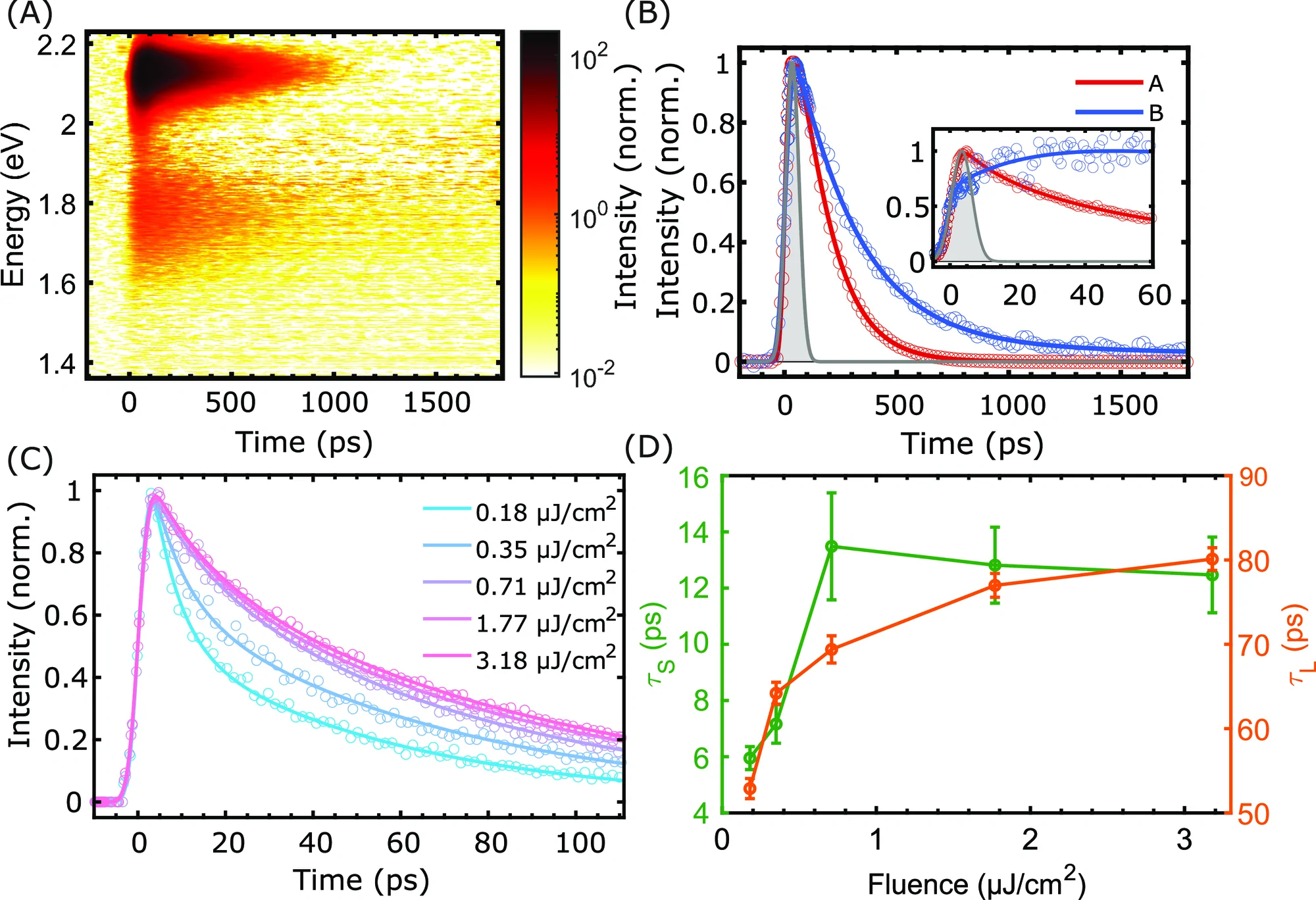
(A) TRPL at 78 K under excitation at 2.43 eV showing an intense peak related to the excitonic feature at 2.14 eV (peak A) and a weaker defect-related emission at 1.8 eV (peak B). The defect emission at 1.3 eV is not observed as it lies outside the sensitivity range of the detector. (B) Normalized decay dynamics of peak A and B along with the corresponding fits. The gray area illustrates the Instrument Response Function. The inset highlights the rise dynamics. (C) Fluence dependence of peak A at 78 K with corresponding fits; higher fluences result in a slower decay dynamics. (D) Results of the biexponential fits to the dynamics in C, showing a clear increase in the time constants with increasing fluences.

The defect-related PL peak exhibits a delayed formation time of approximately 20 ps, followed by a decay with a time constant of the order of hundreds of picoseconds (i.e., ∼300 ps), which is significantly slower than the long-lived decay component of peak A. Crucially, the defect-related emission rises on a time scale comparable to the fast decay component of the excitonic peak. This temporal correlation suggests that defect states quench the peak A emission via exciton capture into localized states, whose recombination occurs at lower energies and with slower dynamics than those of free excitons. Figure 2C shows the temporal dynamics of the emission peak A as a function of excitation fluence at 78 K, while Figure 2D reports the fluence dependence of the decay time constants obtained from the biexponential fitting of traces (τ_*S*_ and τ_*L*_). The longer decay time with increasing fluence is consistent with an increased quantum yield and hence the observed superlinear increase of the PL intensity with fluence (Figure 1D).

The fluence-dependent PL dynamics of peak A differs markedly from those reported, at high excitation densities, in a wide range of semiconductors, including single- and few-layer transition metal dichalcogenides (TMDs), carbon-based low-dimensional systems and 2D quantum wells. In those systems, the decay time typically decreases sharply with increasing photoinduced exciton density, due to exciton–exciton annihilation.^43^ Our results instead highlight the role of defects. Structural imperfections, such as impurities, vacancies, and dislocations, introduce localized electronic states within the bandgap, that act as exciton trapping centers. Trapped excitons can recombine nonradiatively, via defect-assisted pathways, commonly described by the Shockley–Read–Hall (SRH) mechanism. At higher excitation fluences, these defect states progressively saturate as they become filled, suppressing nonradiative recombination channels. Consequently, the relative contribution of radiative processes increases, leading to an effective elongation of the exciton lifetime with increasing fluence. A similar trend has been reported in CVD-grown TMD monolayers, which are typically characterized by a higher defect density.^44–48^ Additional investigations of the TRPL dynamics as a function of temperature have been performed and the results are reported in Figure S5 of the SI. The initial decay of the emission peak A slightly accelerates with increasing temperature, whereas the defect-related dynamics remains largely unchanged.

To gain further insight into the ultrafast dynamics of the material, we performed broadband femtosecond TR measurements. The transient optical response is probed across a broad spectral range spanning not only the optical gap but also higher-energy electronic transitions. Here, the narrowband pump pulse (centered at 2.43 eV) is tuned to match the excitation energy used in the TRPL measurements, whereas the broadband probe covers most of the visible range.

Figure 3A displays the differential reflectivity (Δ*R*/*R*) map measured at 78 K over delay times up to 250 ps. The nonequilibrium optical response is dominated by a pronounced derivative-like feature centered at 2.18 eV, approximately 30 meV above the static excitonic PL peak. This line shape is characteristic of related crystalline systems and arises from the interplay between state filling (Pauli blocking) and bandgap renormalization induced by many-body interactions.^49,50^ Two additional, weaker derivative-like features are observed at higher energies, and are attributed to higher interband transitions (VB to CB + 1 and VB – 1 to CB + 1, see calculations below). The Δ*R*/*R* map is spectrally complex and exhibits both positive and negative signals with opposite signs. These contributions exhibit distinct relaxation dynamics, likely arising from different carrier relaxation and recombination pathways, as well as from many-body interactions that temporally overlap. Furthermore, the low-energy spectral feature shows a gradual blue shift on time scales of several hundred picoseconds, which can be attributed to transient lattice heating followed by cooling.^51^ To disentangle the overlapping spectral components and extract their associated time constants, we performed a global analysis of the Δ*R*/*R* map. This procedure simultaneously fits the full data set, leveraging the fact that different physical processes share common temporal dynamics across probe photon energies. To deconvolute the excited state dynamics, we use a kinetic model of sequentially interconverting Evolution-Associated Spectra (EAS).^52^ Further details on this procedure are provided in the SI. This model assumes a sequence of monoexponential decays with increasing time constants τ_*i*_, which represent the lifetime of each spectral component (Figure 3B). The analysis yields three distinct components. The first two EAS (τ_1_ and τ_2_) exhibit nearly identical spectral signatures, characterized by derivative-like features at the interband transition energies, while the third component is localized within the bandgap region and decays on a much longer time scale (τ_3_). The extracted decay times associated with the EAS spectra for the lowest fluence are τ_1_ = 1.48 ± 0.02 ps, τ_2_ = 17.75 *±* 0.02 ps, and τ_3_ > 300 ps, respectively. Similar time scales have been reported in other layered semiconductors such as TMDs. Based on these time scales, we assign τ_1_ to electron–phonon scattering: above bandgap photoexcitation at 2.43 eV creates a population of hot electrons and holes in the conduction and the valence bands well above the band edges, which rapidly relax via phonon emission. The intermediate time scale τ_2_ is attributed to carrier trapping at defect states, in quantitative agreement with the rise time of the defect states emission extracted from the complementary TRPL measurements. The longest decay component τ_3_ extending over hundreds of picoseconds can be attributed to nonradiative trap-assisted recombination of the excited carriers; however, on these time scales, a contribution from transient lattice heating cannot be excluded.

**Figure 3:**
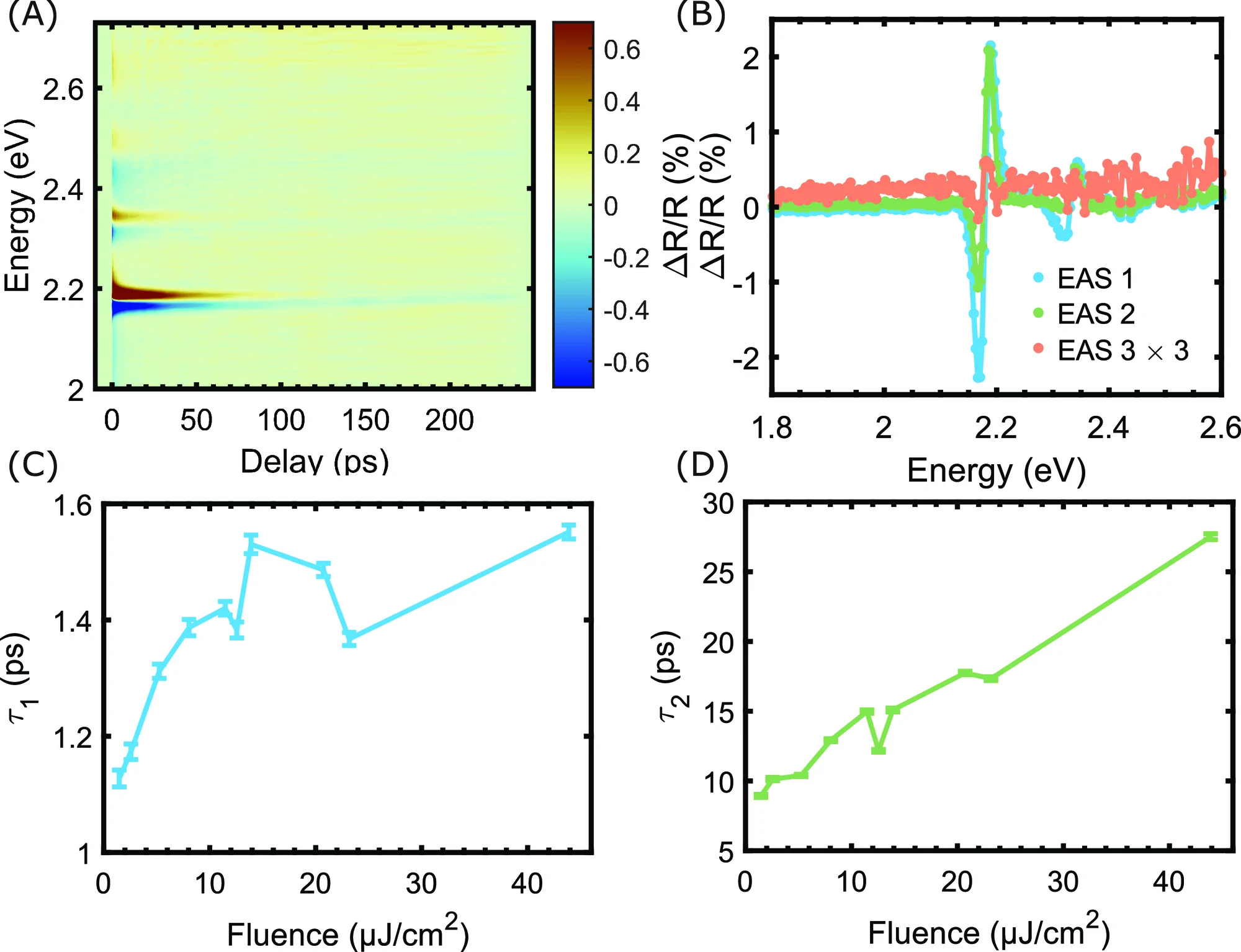
(A) TR map at 78 K. The transient response displays three distinct derivative-like features, at 2.19, 2.35, and 2.5 eV, respectively. We assign these features to pump-induced modification of interband transitions observed in the optical response of the material. (B) Evolution-Associated Spectra (EAS) obtained from sequential global analysis using a three-component kinetic model. The first two components (EAS 1, EAS 2) are dominated by high-energy transitions (2.35 and 2.47 eV), while EAS 3 is confined at the band edge. (C, D) Fluence dependence of the extracted time constants τ_1_ and τ_2_, respectively. Error bars represent the standard errors of the fit.

We further investigate the fluence dependence of these dynamics, as summarized in Figure 3C and D. Both τ_1_ and τ_2_ exhibit a clear increase with increasing pump fluence, indicating a progressive slowing down of the carrier relaxation dynamics at higher excitation densities. The fast component τ_1_ increases by approximately ∼50% across the explored fluence range. A possible mechanism underlying this behavior is a phonon bottleneck:^53,54^ at increasing excitation density, the nonequilibrium phonon population generated during hot-carrier relaxation increases, enhancing phonon reabsorption and thereby reducing the efficiency of carrier cooling. However, state-filling effects may also contribute to the observed slowing down, as the progressive occupation of electronic states accessible to relaxing hot carriers reduces the available phase space for scattering through Pauli blocking. The second time constant, τ_2_, occurs on a time scale comparable to the slow dynamics observed in the TRPL measurements and also exhibits a pronounced increase with fluence, with its value approximately tripling over the investigated fluence range. This behavior is consistent with the progressive filling and saturation of sub-bandgap states involved in carrier localization, which reduces the efficiency of defect-assisted relaxation channels at high excitation densities. Overall, the fluence-dependent dynamics therefore point to the coexistence of multiple density-dependent relaxation mechanisms, including carrier localization into sub-bandgap states, state filling, and phonon-bottleneck-limited carrier cooling.

In order to establish the electronic landscape underlying the optical response of HgPSe_3_ and to rationalize the energy mismatch between the TR and TRPL signals near the optical gap, we performed electronic structure calculations along the high-symmetry path of the Brillouin zone.

The electronic band structure of HgPSe_3_ was calculated within DFT using the Perdew–Burke–Ernzerhof (PBE) functional,^17^ including vdW Grimme D3 corrections^20^ and spin–orbit coupling (SOC).

The orbital-projected electronic structure provides a microscopic foundation for interpreting the near-edge optical response. The valence band maximum is primarily composed of Se *p* orbitals and is located at the Brillouin-zone center, Γ. The conduction band edge exhibits a markedly different character: its lowest states originate from strong hybridization between Hg *s* orbitals and Se *p* orbitals. As a result of this hybridization, the conduction band minimum is displaced from Γ and forms an off-center valley along the Γ–V direction, hereafter denoted as *V*_*A*_.

A systematic analysis of the conduction-band dispersion demonstrates that *V*_*A*_ constitutes the global minimum of the conduction band. In particular, two-dimensional energy maps around Γ, evaluated for several *k*_*z*_ planes, reveal that no lower-energy conduction band states occur elsewhere in the Brillouin zone (see SI, Figure S8). Therefore, while the band edge is not strictly located at Γ, the small momentum offset and the relatively flat dispersion around *V*_*A*_ place this valley in close energetic and reciprocal-space proximity to the direct Γ transition.

In broadband TR experiments, the optical response is dominated by interband transitions, and thus by direct band-to-band processes in the electronic band structure. For this reason, the comparison between theory and experiment is performed in terms of direct transitions extracted from the calculated band structure. To enable a meaningful comparison, a scissor correction is applied to the PBE+vdW+SOC electronic structure (Figure 4B), aligning the lowest-energy direct transition with the experimentally observed near-edge resonance. This step is essential for two closely related reasons. First, an accurate positioning of the quasiparticle gap is a prerequisite for any quantitative treatment of excitonic effects, as the relative separation between bound excitonic states and the continuum depends sensitively on the underlying single-particle spectrum. Second, the scissor-corrected band structure enables a clear identification of higher-lying direct interband transitions with substantial optical oscillator strength. In particular, the scissor-corrected DFT calculations yield optically active direct transitions with large dipole matrix elements at 2.19, 2.31, and 2.43 eV, corresponding respectively to the VB *→* CB transition at the *V*_*A*_ valley and to the VB *→* CB + 1 and VB*–*1 *→* CB + 1 transitions at Γ (Figure 4C). The dipole matrix elements were evaluated within density functional perturbation theory (DFPT), ensuring a consistent description of the optical transition strengths. These energies are in good agreement with the three derivative-like features observed in the broadband TR spectra at approximately 2.2, 2.35, and 2.5 eV. Given that the present DFT calculations describe the electronic structure at 0 K, while the TR measurements were performed at finite temperature, the observed level of agreement in both energy scale and relative spacing of the transitions is fully sufficient and physically meaningful for a comparison between theory and experiment.

**Figure 4:**
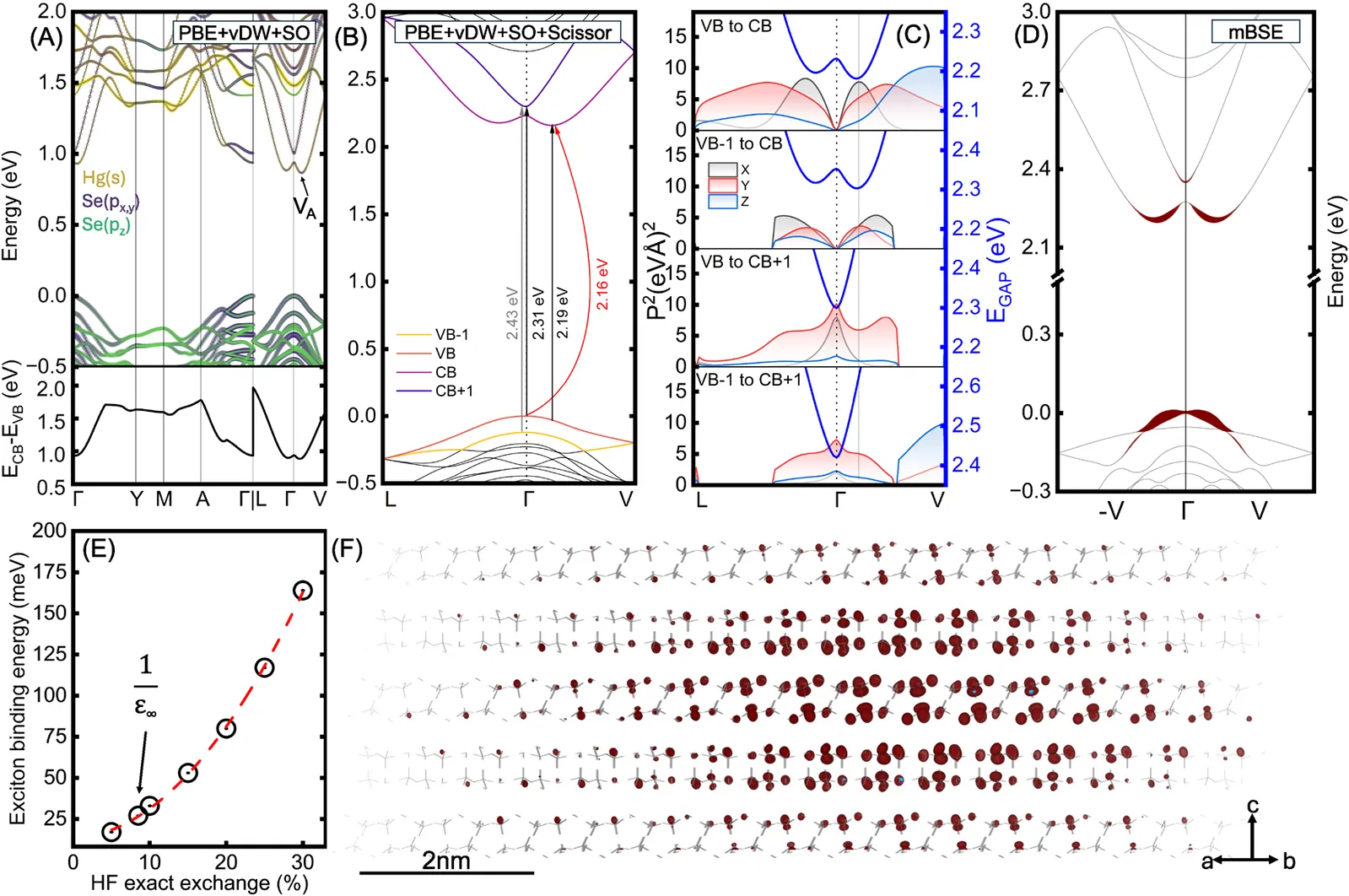
(A) Orbital-projected band structure of HgPSe_3_ calculated within PBE+vdW+SOC, highlighting the off-Γ conduction-band valley *V*_*A*_. The lower panel shows the band gap variation along the high-symmetry path. (B) Scissor-corrected band structure with the relevant interband transitions indicated. (C) Squared dipole matrix elements for different band combinations along the L−Γ–V path and for different light polarizations. (D) Reciprocal-space projection of the lowest bright excitonic state obtained from mBSE calculations. (E) Binding energy of the lowest bright exciton as a function of the Hartree–Fock exact-exchange fraction. (F) Real-space excitonic wave function for the lowest bright exciton (hole fixed in the vicinity of a Se site).

The excitonic calculations were primarily motivated by the need to rationalize the PL peak observed at energies slightly below the lowest resonance detected in the broadband TR spectra. Two physical scenarios can, in principle, account for this behavior. The first scenario corresponds to a quasi-direct band-to-band transition involving the off-Γ valley *V*_*A*_, whose close proximity to the Γ point and relatively flat dispersion enhance the joint density of states. This interpretation is energetically consistent, as the scissor-corrected DFT places the lowest direct transition at approximately 2.16 eV, in excellent agreement with the PL peak observed at ∼2.14 eV. An alternative interpretation of the near-band-edge PL involves the formation of a free excitonic state lying slightly below the band-edge continuum. To examine this possibility, excitonic effects were investigated using time-dependent density functional theory within the model Bethe–Salpeter equation (mBSE) formalism.^22,23^ This approach enables an explicit treatment of electron–hole interactions beyond the independent-particle picture and is particularly suited for assessing the nature and spatial character of excitonic states.

It is well established that mBSE calculations are sensitive to the choice of model parameters, most notably to the fraction of exact Hartree–Fock (HF) exchange entering the electron–hole interaction kernel (details of method including convergence tests are described in the SI and in Figure S9). Within a physically reasonable range of these parameters, however, the results are remarkably consistent. In all cases, the lowest bright excitonic state (located at the *V*_*A*_ valley) is characterized by a relatively small binding energy and by a spatially extended wave function with pronounced multilayer character (Figure 4F). Within the dielectric-dependent hybrid (DDH) schemes (where HF is equal to the reciprocal of the dielectric constant), the binding energy of the lowest exciton amounts to approximately 27 meV.

These findings indicate that, even when excitonic effects are explicitly included, the resulting exciton is weakly bound and only marginally separated from the band-edge continuum. Consequently, such an excitonic state could reasonably appear in the vicinity of the experimentally observed PL energy around 2.14 eV, but it is not expected to manifest as a sharply isolated excitonic resonance. Rather, its delocalized nature suggests that excitonic effects primarily act to renormalize and reshape the optical response associated with the band edge.

Taken together, the mBSE results are fully consistent with a quasi-direct band-edge scenario as the dominant origin of the near-band-edge PL; this conclusion is robust with respect to the rigid scissor correction, which affects the absolute transition energies but not the band ordering, relative transition separations, dipole matrix elements, or the location of the VA valley. At the same time, they indicate that weakly bound excitonic effects may provide a non-negligible contribution to the optical response, particularly in the presence of a near-degenerate indirect valley VA and enhanced joint density of states, although the numerical exciton binding energy depends on the adopted HF fraction and screening model. This interplay naturally reconciles the close energetic correspondence between the PL peak and the lowest direct transition observed in the TR spectra, while the relative contributions of quasi-direct band-to-band and excitonic recombination cannot be uniquely determined.

## Conclusions

We obtained a comprehensive picture of the exciton and carrier dynamics in HgPSe_3_, highlighting the interplay between carrier localization, sub-bandgap emission, and phonon-mediated relaxation. In addition to the excitonic emission near 2.1 eV and the previously reported sub-bandgap feature around 1.8 eV, we identify a lower-energy emission centered at approximately 1.3 eV. The distinct temperature and fluence dependences of these sub-bandgap features^8,10,38^ point to the involvement of different localized electronic states and relaxation pathways. TRPL measurements reveal the progressive population of long-lived localized states following photoexcitation, providing direct information on the time scales governing the redistribution and recombination of photoexcited carriers. Broadband transient-reflectivity measurements complement this picture by resolving multiple relaxation components associated with the evolution of the photoexcited carrier population.^46–48,50^ Their fluence dependence indicates that several density-dependent processes contribute to the relaxation dynamics. In particular, the slowing down of the fast component at increasing excitation density is consistent with a hot-phonon bottleneck, although state-filling effects may also contribute, while the intermediate dynamics are compatible with carrier localization into sub-bandgap states. At longer delays, both long-lived carrier populations and transient lattice heating may contribute to the optical response. Overall, our results reveal a complex interplay between carrier relaxation, localization, and electron–phonon interactions in HgPSe_3_ and establish a framework for understanding its nonequilibrium optical response. The coexistence of visible interband transitions and long-lived sub-bandgap optical responses may be relevant for future studies exploring HgPSe_3_ and related MPX_3_ semiconductors for broadband optoelectronic and photodetection applications.

## Supplementary Material



## Notes

Analysis of temperature-dependent bandgap renormalization, temperature- dependent TRPL signals, and the conduction band energy landscape. Description of the fitting of TRPL dynamics and global fitting of the Δ*R*/*R* maps; temperature dependence of the optical bandgap with Varshni relation fit (Figure S1); evolution of static photoluminescence spectra (Figure S2); temperature-dependent peak positions for Peak A, B and C (Figure S3); Arrhenius analysis and activation energy fit for Peak C (Figure S4); TRPL decay dynamics for Peaks A and B (Figure S5); fluence dependence of fits of Peak A dynamics (Figure S6); comparison of global fitting of Δ*R*/*R* maps and experimental data (Figure S7); 2D conduction band energy maps across different *k*_*z*_ planes and Brillouin zone representation (Figure S8); and convergence analysis of exciton binding energy and dielectric constants (Figure S9) (PDF)
