# Supplementary material for: The Interplay of Phonons and Subgap States Governs Ultrafast Carrier Dynamics in Layered HgPSe3
Source: J Phys Chem C Nanomater Interfaces. 2026 Oct 1;130(41):14220–9. doi: 10.1021/acs.jpcc.6c03448 (PMC13679094; doi:10.1021/acs.jpcc.6c03448)
Supplement: Supplementary file 1 [file jp6c03448_si_001.pdf]

Supporting information for:

The interplay of phonons and sub-gap states governs ultrafast carrier dynamics in layered $HgPSe_3$

Alessandro Baserga[1], Milosz Rybak[2], Micol Bertolotti[1], Antonella Treglia[3], Samim Sardar[3], Valentino Jadriško[1], Matteo Ghezzi[1], Giuseppe Maria Paternò[1], Cosimo D'Andrea[3], Annamaria Petrozza[3], Zdenek Sofer[4], Artur P. Herman[2], Robert Kudrawiec[2], Giulio Cerullo[1], Giulia Folpini[3], Nikolas Antonatos[2], Christoph Gadermaier[1,*], and Stefano Dal Conte[1,*]

[1]Department of Physics, Politecnico di Milano, Piazza L. da Vinci 32, 20133 Milano, Italy
[2]Department of Semiconductor Materials Engineering, Wrocław University of Science and Technology, Wybrzeże Wyspiańskiego 27, 50-370 Wrocław, Poland
[3]Istituto Italiano di Tecnologia, Milano & 20134, Italy
[4]Department of Inorganic Chemistry, University of Chemistry and Technology Prague, Technická 5, 166 28 Prague 6, Czech Republic
*Email: christoph.gadermaier@polimi.it, stefano.dalconte@polimi.it

The PDF file includes:

- **Supporting text:** Details on temperature-dependent bandgap renormalization, fitting procedures for TRPL dynamics and $\Delta R/R$ maps, and the conduction band energy landscape.
- **Supporting figures (Figures S1 to S9):** Data covering static and time-resolved photoluminescence, Arrhenius activation analysis, kinetic fit comparisons, and theoretical convergence tests.

Supporting text

1 Further Experimental Characterizations

Figure S1 displays the temperature dependence of the optical bandgap of $HgPSe_3$, extracted from the Tauc analysis of the static absorption spectra. To quantify the renormalization of the band structure due to electron-phonon interactions and lattice expansion, the experimental data were fitted using the semi-empirical Varshni relation:

$$E_g(T) = E_g(0) - \frac{\alpha T^2}{T + \beta} \tag{S1}$$

where $E_g(0)$ is the bandgap energy at absolute zero, α represents the high-temperature slope of the bandgap shift, and β is a parameter related to the Debye temperature of the material. The best fit is obtained with the values $E_g(0) = 2.167 \pm 0.004\,\mathrm{eV}$, $\alpha = (7.42 \pm 0.39) \times 10^{-4}\,\mathrm{eV/K}$, and $\beta = 54 \pm 21\,\mathrm{K}$. These indicate a significant electron-phonon coupling strength.

Figure S2 presents the temperature-dependent static photoluminescence (PL) spectra of $HgPSe_3$. The evolution of Peak A is clearly visible, showing a monotonic blue shift in energy and a significant increase in intensity as the temperature decreases, consistent with temperature-dependent bandgap renormalization and conventional thermal quencing. At the same time, the interplay between the defect-related emissions (Peaks B and C) can be appreciated. Peak B (centered at $\approx 1.8\,\mathrm{eV}$) emerges only at low temperatures (T < 123 K) and its intensity increases as the temperature is lowered. In contrast, Peak C (centered at $\approx 1.3\,\mathrm{eV}$) displays a non-monotonic behavior, reaching its maximum intensity at an intermediate temperature of approximately 170 K before being quenched at both higher and lower temperatures.

Figure S3 summarizes the temperature dependence of the peak energy positions for the three emission features identified in the static PL spectra (Figure S2). Peak A exhibits a redshift with increasing temperature, following the renormalization of the bandgap. Peaks B and C remain nearly constant over the entire temperature range. This spectral stability is characteristic of intragap defect states, which are less sensitive to lattice expansion and phonon-induced band edge shifts.

Figure S4 illustrates the Arrhenius analysis of the temperature-dependent intensity of the defect-related photoluminescence peak C. To determine the thermal activation energy required for carrier detrapping or non-radiative quenching, the integrated intensity was fitted using the relation:

$$I(T) = I_0 \exp\left(\frac{E_a}{k_B T}\right) \tag{S2}$$

where I_0 is a pre-exponential factor, k_B is the Boltzmann constant, and E_a represents the activation energy of the defect state [1]. The best fit to the experimental data yields an activation energy of $E_a = 71 \pm 12\,\mathrm{meV}$ and a pre-factor of $I_0 = 3.5 \pm 2.5$.

Figure S5 illustrates the temperature dependence of the time-resolved photoluminescence (TRPL) decay dynamics for Peaks A and B, measured from 78 K to 300 K at a constant excitation power of 50 µW. Slightly faster dynamics are observed at higher temperatures for peak A, while no significant changes are observed for peak B.

2 Fitting of TRPL dynamics

The extracted dynamics of peaks A and B were fitted using a biexponential decay convoluted with a Gaussian instrument response function (IRF). The signal $y(t)$ is described by the analytical expression $y(t) = \sum_{i=1}^{2} \frac{A_i}{2} \exp\left(\frac{\tau_{\mathrm{IRF}}^2}{2\tau_i^2} - \frac{t-t_0}{\tau_i}\right)\left[1 + \mathrm{erf}\left(\frac{t-t_0-(\tau_{\mathrm{IRF}}^2/\tau_i)}{\tau_{\mathrm{IRF}}\sqrt{2}}\right)\right]$, where A_i and τ_i are the amplitude and time constant of the i-th decay component, t_0 is the time zero shift, and τ_{IRF} represents the width of the Gaussian IRF. The shorter of the two fitted components is relabeled as S for short and the second as L, which stands for long. The resulting fits accurately reproduce

the experimental data, confirming an increase in both characteristic time constants (τ_S and τ_L). Figure S6 illustrates the relative contributions of the two exponential terms, demonstrating that the weight of the second, slower decay component becomes increasingly dominant, and confirms that the fitted τ_{IRF} does not significantly change across the measurements.

3 Global Fitting of $\Delta R/R$ maps

Given the complexity of the transient optical response of $HgPSe_3$, where different relaxation processes may contribute with different spectral weights across the probed energy range, we employed a global analysis of the complete time-resolved spectral dataset. The analysis was performed using Glotaran[2, 3], adopting a purely sequential, unbranched kinetic model, an approach previously employed for the analysis of ultrafast dynamics in semiconductor and halide-perovskite nanostructures[4–6]. Within this framework, the experimental data matrix is decomposed into a set of Evolution Associated Spectra (EAS) and their corresponding characteristic timescales. Each EAS describes the transient spectral response associated with a given stage of the temporal evolution defined by the kinetic model and does not necessarily represent a distinct microscopic excited state. In the sequential model adopted here, the first EAS describes the transient optical response established immediately after photoexcitation, while the subsequent EAS components describe successive stages of the relaxation dynamics, from the shortest to the longest characteristic timescale. We stress that this sequential scheme provides a phenomenological description of the experimental dynamics and should not be interpreted as a unique microscopic relaxation pathway. Nevertheless, in Figure S7, we provide a direct comparison between the experimental pump–probe temporal traces acquired at selected probe energies corresponding to the derivative-like spectral features of the interband transitions and the temporal dynamics reconstructed from the Glotaran global analysis. The good agreement between the experimental and reconstructed traces demonstrates that the global fitting procedure accurately captures the measured transient dynamics. Finally, all the extracted timescales, including the shortest one, are longer than the temporal resolution of the pump–probe setup (approximately 100 fs), indicating that their determination is not significantly limited by the finite instrument response.

4 Conduction Band Energy Landscape

To verify the nature of the bandgap and the precise location of the conduction band minimum (CBM), we performed a systematic mapping of the electronic dispersion across the Brillouin zone. Figure S8 displays two-dimensional energy contour plots of the lowest conduction band in the vicinity of the Γ point, evaluated at various k_z planes ranging from $k_z = 0$ to $k_z = 0.5$ (in units of reciprocal lattice vectors).

The calculations reveal that the global energy minimum does not occur strictly at the high-symmetry point Γ, but rather at an off-center valley, denoted as V_A, located along the Γ–Y direction. As shown in the energy maps, this valley forms a shallow potential well that is energetically lower than the states at Γ. Crucially, an extensive search throughout the entire Brillouin zone confirms that no other valleys with lower energy exist, establishing V_A as the true CBM. Despite this momentum offset, the energy difference between V_A and the direct transition at Γ is minimal, and the dispersion remains relatively flat. This proximity in both energy and reciprocal space imparts a "quasi-direct" character to the optical transitions, consistent with the strong absorption edge observed experimentally.

Model dielectric function

The exciton binding energies were evaluated using the Bethe–Salpeter equation (BSE) in its model form (mBSE)[7, 8], where the screened Coulomb interaction is approximated by a model

dielectric function of the form:

$$\varepsilon^{-1}(\mathbf{q}+\mathbf{G}) = 1 - (1 - H_{\mathrm{F}}) \exp\left[-\frac{2\pi|\mathbf{q}+\mathbf{G}|^2}{4\lambda}\right], \tag{S3}$$

where H_{F} is the inverse effective macroscopic dielectric constant obtained from DFPT (averaged in this work), and λ is a screening-length parameter. This expression renders the screened interaction diagonal in reciprocal space and allows efficient convergence of excitonic states with respect to k-point sampling.

As shown in Fig.S9A, the convergence of the first bright exciton binding energy with respect to the k-mesh is slow. Extrapolation to the infinite-mesh limit [Fig.S9B] yields robust values, consistent with previously reported trends. [9]

The dielectric constant entering the screening was computed in two ways: (i) using the frequency-dependent dielectric function obtained from interband optical transitions (`LOPTICS`), and (ii) using the static dielectric tensor from DFPT linear-response to homogeneous electric fields (`LEPSILON`).

The exciton binding energy is finally obtained as the difference between the independent-particle transition energy and the first bright BSE eigenvalue.

Supporting figures

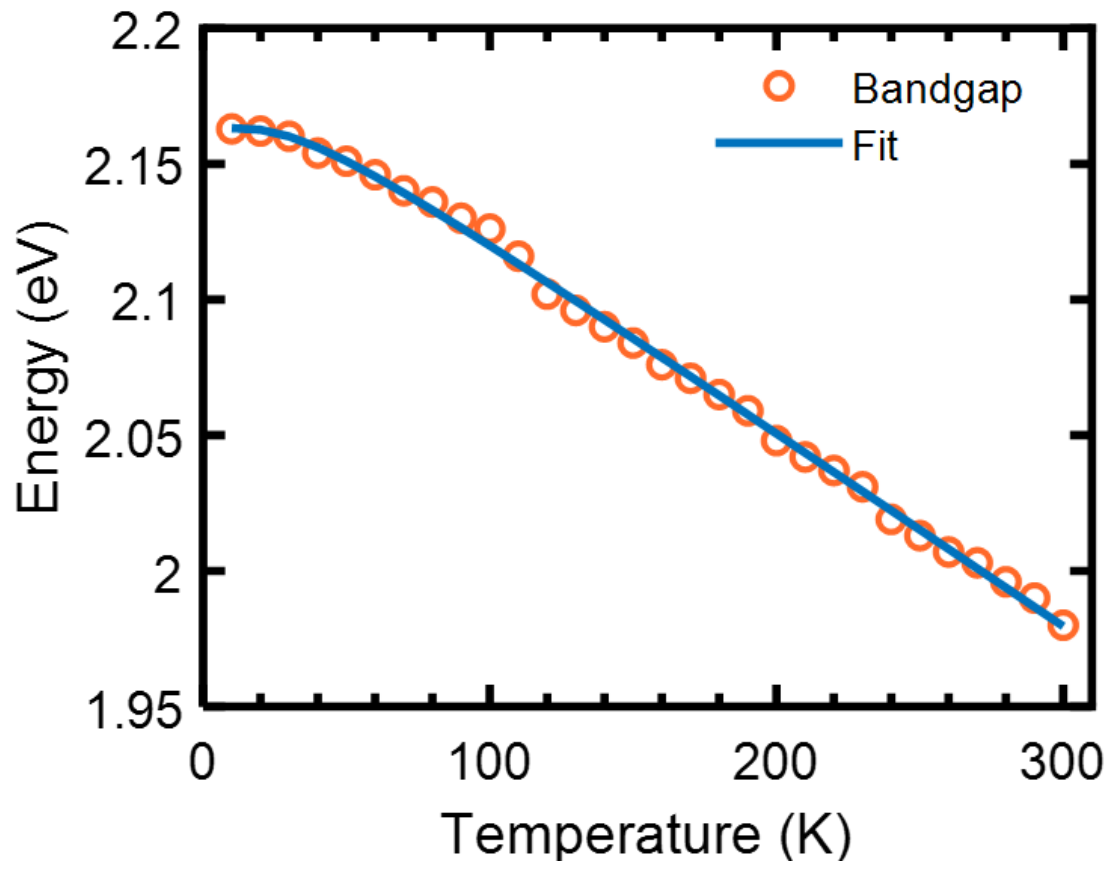

Figure S1: Temperature dependence of the bandgap and Varshni relation fit

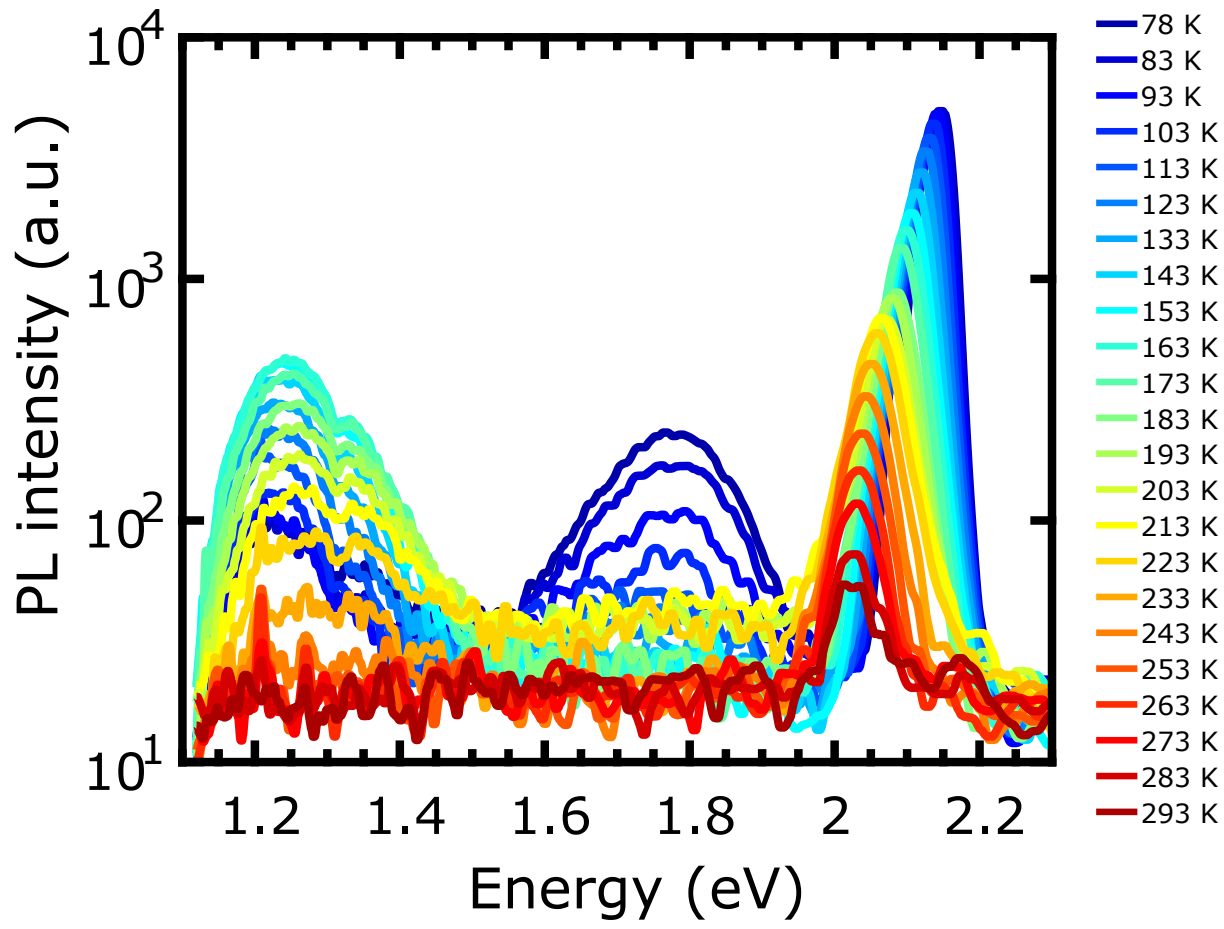

Figure S2: Temperature dependence of PL spectrum

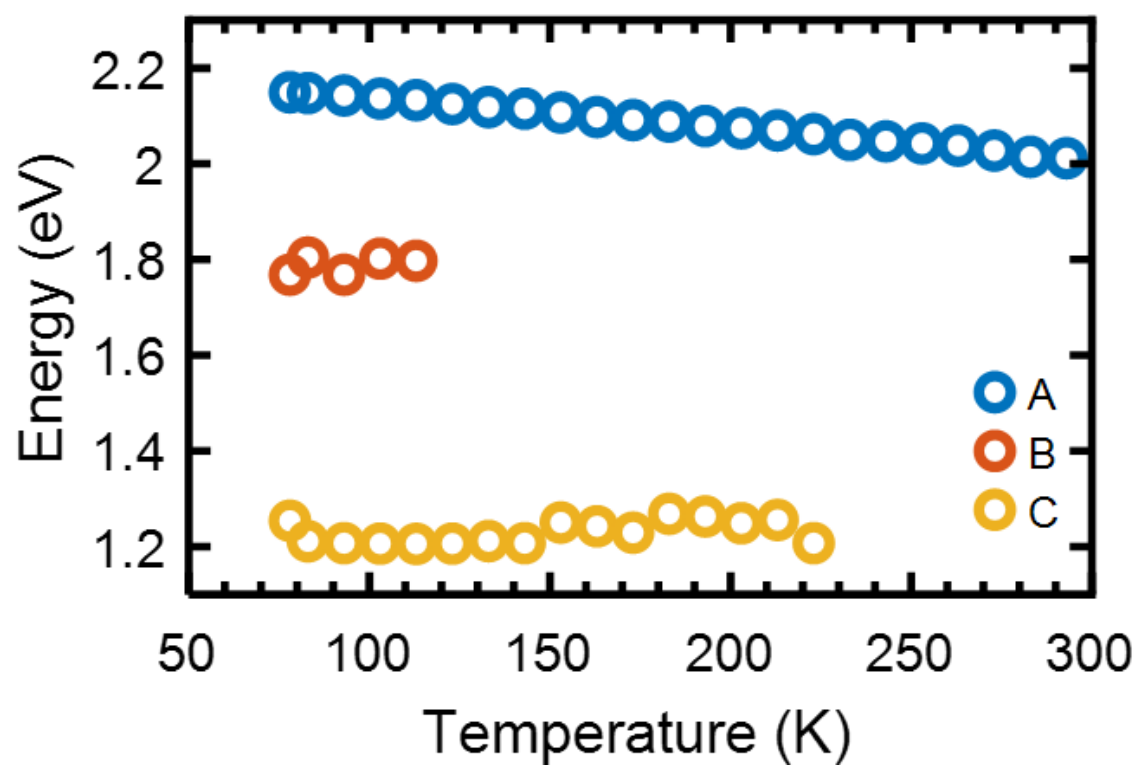

Figure S3: Temperature dependence of the PL peak energies

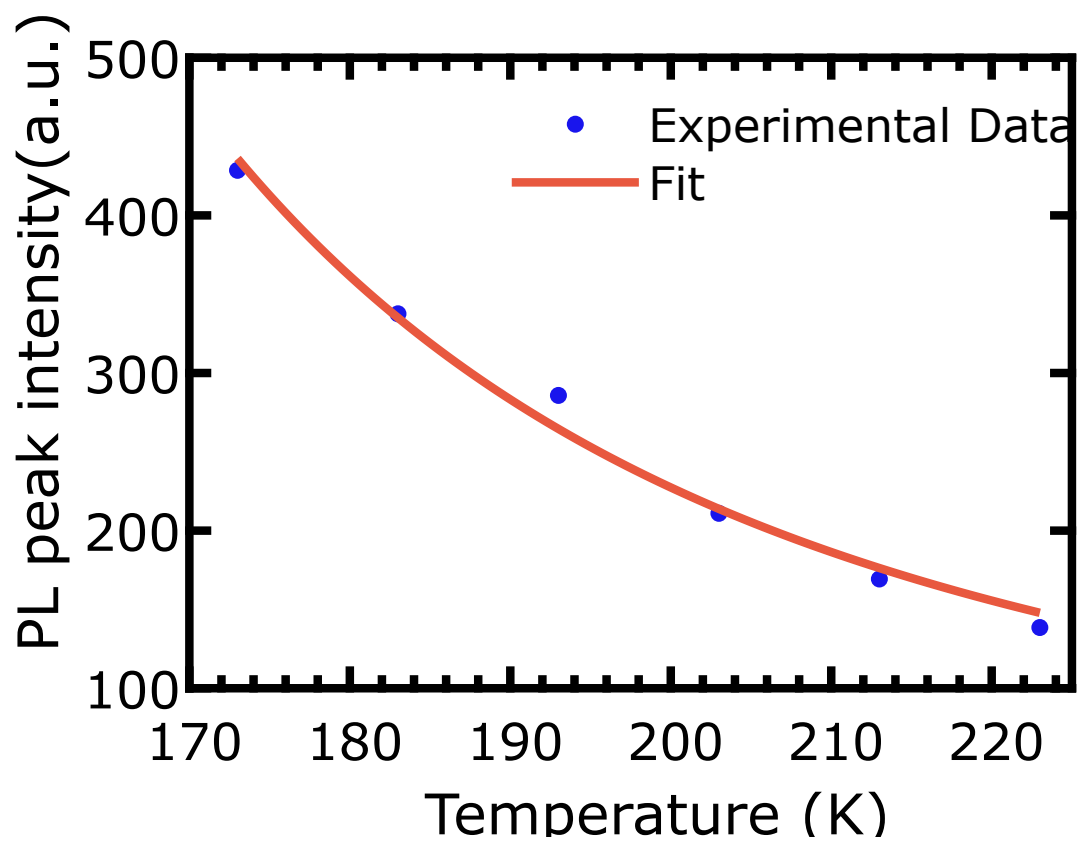

Figure S4: Temperature dependence of PL peak intensity of peak C in selected temperature range and activation energy fit

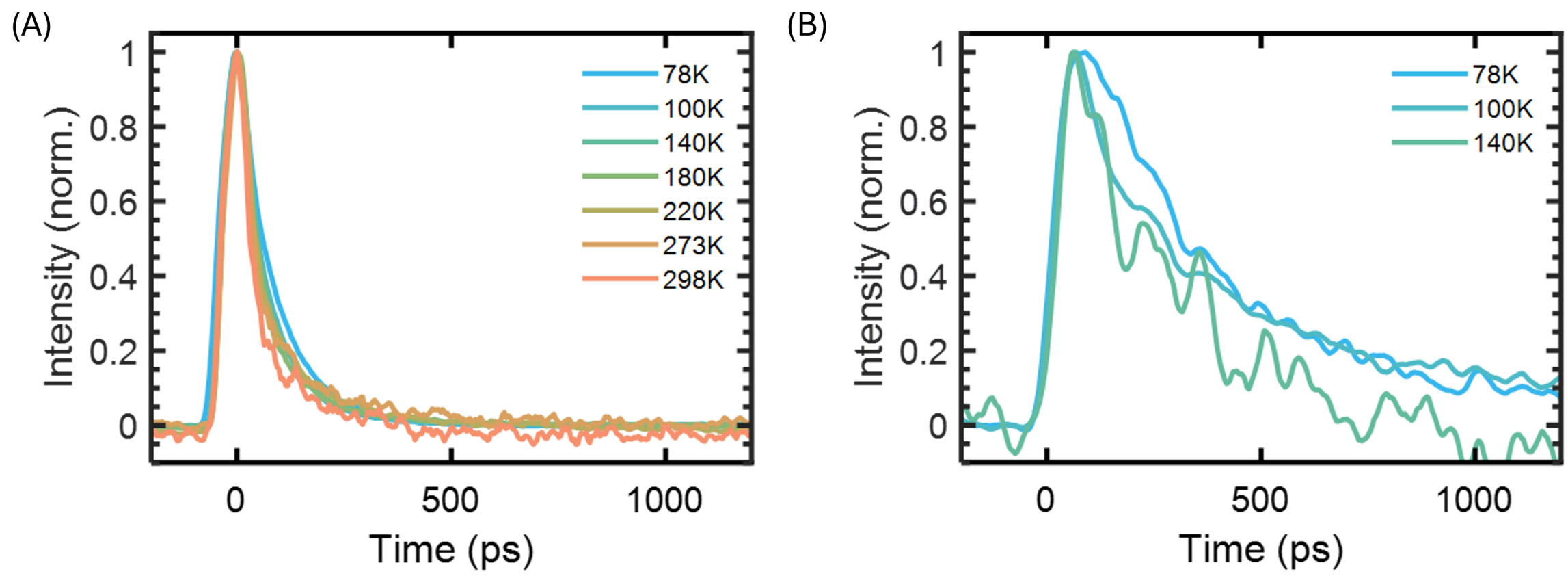

Figure S5: (A) Temperature dependence of the dynamics of peak A (B) Temperature dependence of the dynamics of peak B

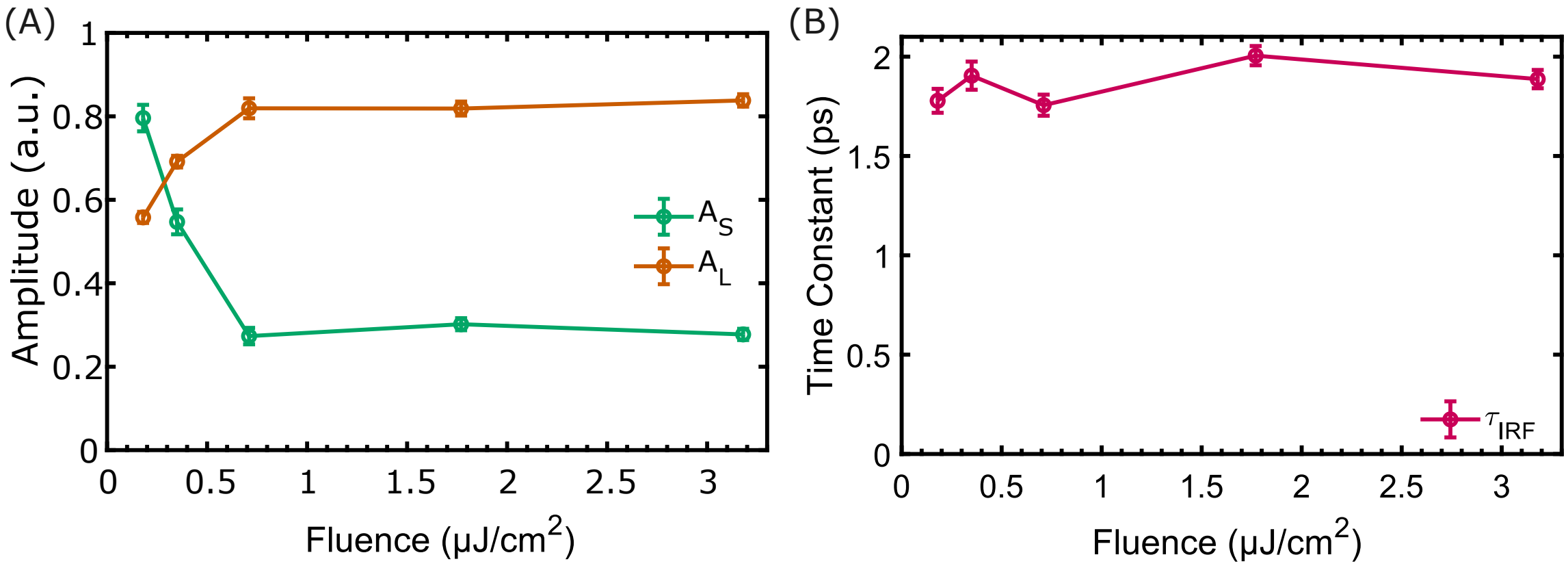

Figure S6: (A) Relative contributions of the two exponential terms extracted from the biexponential fits of the TRPL dynamics for peaks A and B. The data demonstrate that the relative weight of the second, slower exponential component becomes increasingly dominant. (B) The fitted τ_{IRF} is also presented, confirming that it does not significantly change across the measurements.

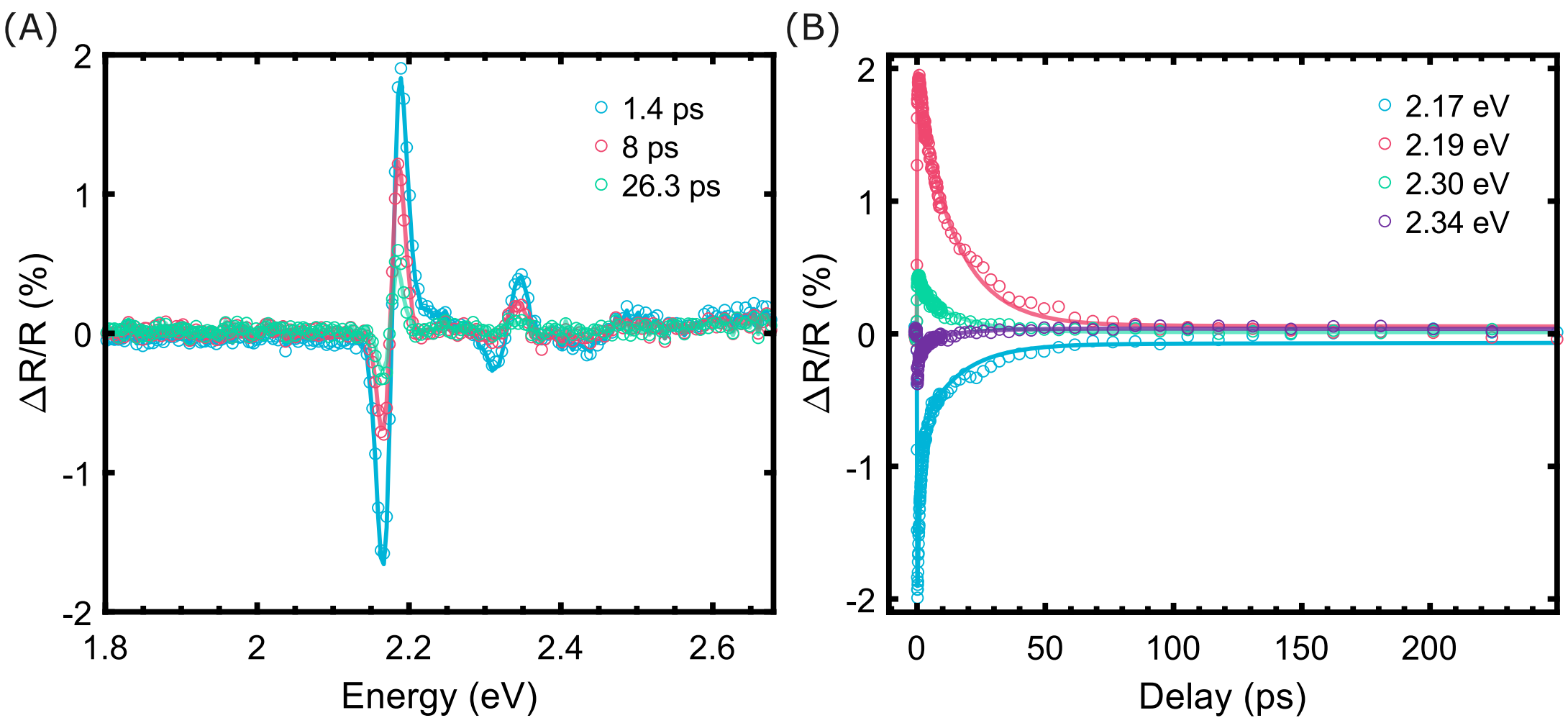

Figure S7: (A) Spectra measured at an incident pump fluence of 11.4 μJ/cm^2. The experimental data are represented by points, and the solid lines denote the results of the global fit. (B) Time-resolved decay dynamics at the minimum and maximum of the two lower derivative shapes corresponding to the first two interband transitions for the same excitation fluence. Points indicate the experimental measurements, while the solid lines show the corresponding global fit obtained via the sequential kinetic model.

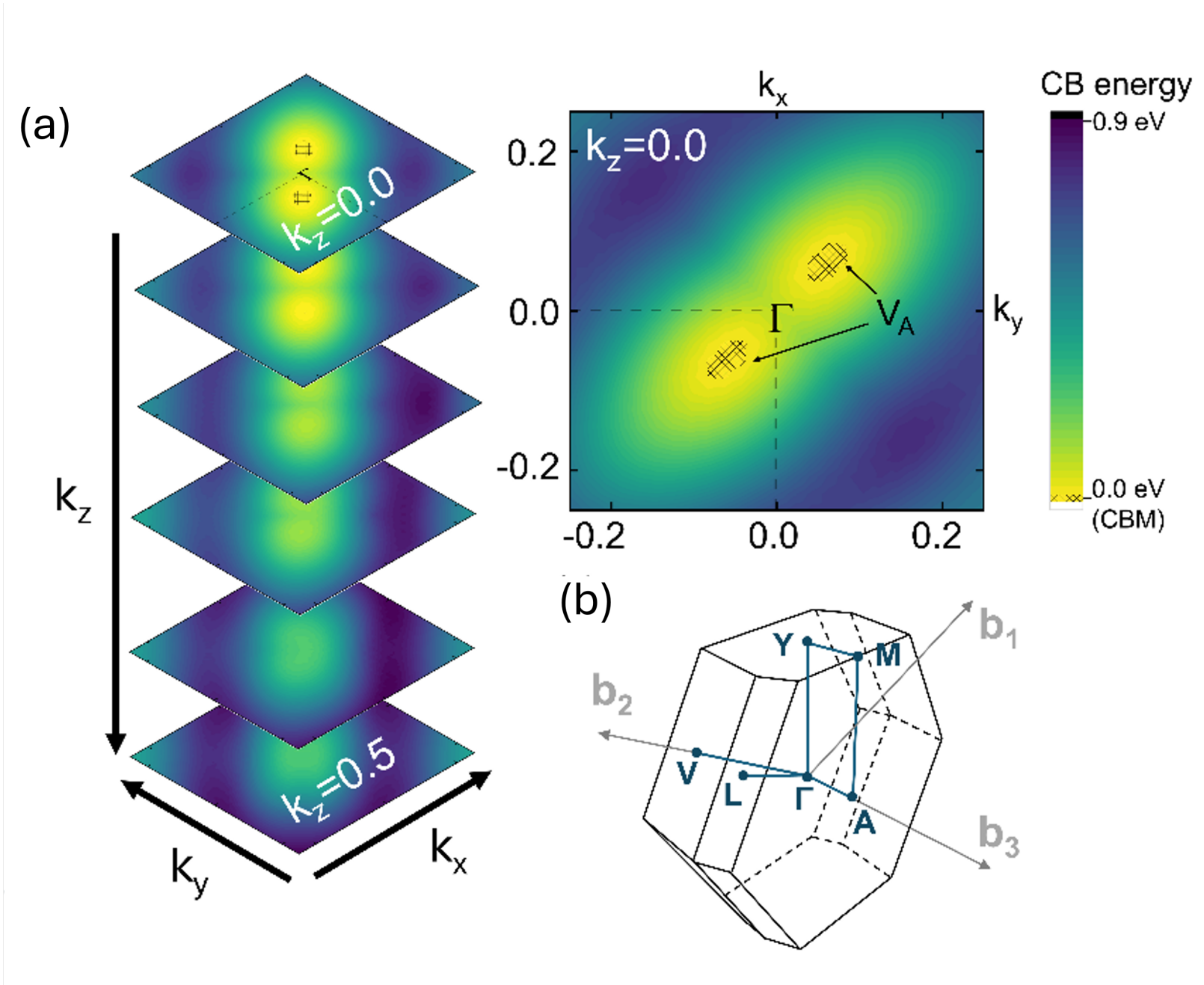

Figure S8: (a) Conduction band energy maps around Γ for different k_z planes (b) First Brillouin zone of Cn/2m bulk $HgPSe_3$.

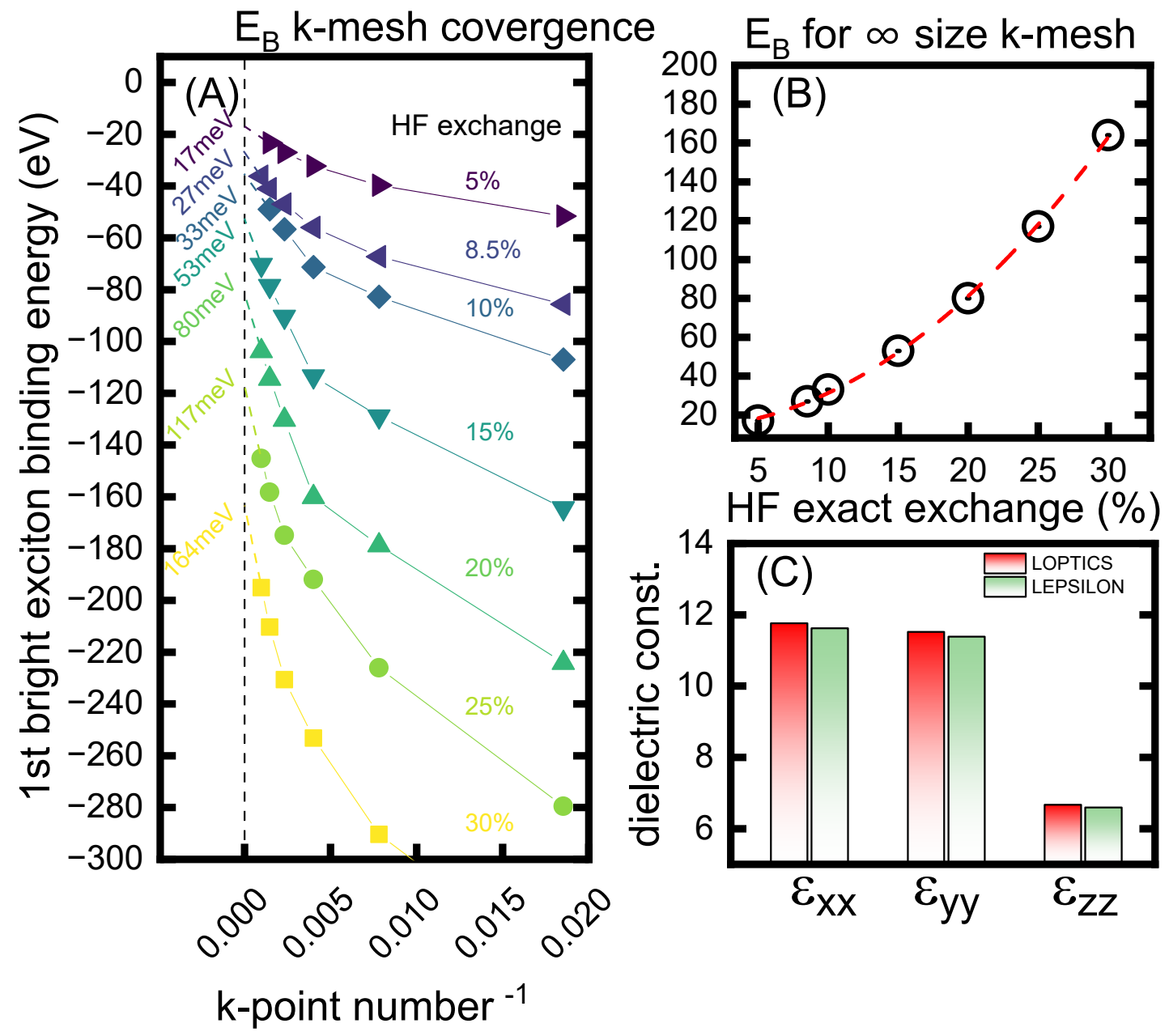

Figure S9: (A) Convergence of the first bright exciton binding energy with k-point sampling. (B) Binding energy as a function of Hartree–Fock fraction. (C) Dielectric constants obtained with `LEPSILON` and `LOPTICS`.

References

[1] de Simoni, B.; Rybak, M.; Antonatos, N.; Herman, A. P.; Ciesiołkiewicz, K.; Tołłoczko, A. K.; Peter, M.; Piejko, A.; Mosina, K.; Sofer, Z.; Kudrawiec, R. Electronic Band Structure and Optical Properties of $HgPS_3$ Crystal and Layers. *The Journal of Physical Chemistry C* **2024**, *128*, 9270–9280.

[2] Snellenburg, J. J.; Laptenok, S. P.; Seger, R.; Mullen, K. M.; van Stokkum, I. H. M. Glotaran: A Java-Based Graphical User Interface for the R Package TIMP. *Journal of Statistical Software* **2012**, *49*.

[3] van Stokkum, I. H.; Larsen, D. S.; van Grondelle, R. Global and target analysis of time-resolved spectra. *Biochimica et Biophysica Acta (BBA) - Bioenergetics* **2004**, *1657*, 82–104.

[4] Toyota, Y.; Sagawa, M.; Yamashita, S.; Okayasu, Y.; Nagai, Y.; Okada, Y.; Kobayashi, Y. Effect of the bulkiness of alkyl ligands on the excited-state dynamics of ZnO nanocrystals. *RSC Advances* **2024**, *14*, 2796–2803.

[5] Franco, C. V.; Mahler, B.; Cornaggia, C.; Gustavsson, T.; Cassette, E. Charge Carrier Relaxation in Colloidal $FAPbI_3$ Nanostructures Using Global Analysis. *Nanomaterials* **2020**, *10*.

[6] Slavov, C.; Hartmann, H.; Wachtveitl, J. Implementation and Evaluation of Data Analysis Strategies for Time-Resolved Optical Spectroscopy. *Analytical Chemistry* **2015**, *87*, 2328–2336.

[7] Sander, T.; Maggio, E.; Kresse, G. Beyond the Tamm-Dancoff approximation for extended systems using exact diagonalization. *Phys. Rev. B* **2015**, *92*, 045209.

[8] Tal, A.; Liu, P.; Kresse, G.; Pasquarello, A. Accurate optical spectra through time-dependent density functional theory based on screening-dependent hybrid functionals. *Phys. Rev. Res.* **2020**, *2*, 032019.

[9] Bokdam, M.; Sander, T.; Stroppa, A.; Picozzi, S.; Sarma, D. D.; Franchini, C.; Kresse, G. Role of Polar Phonons in the Photo Excited State of Metal Halide Perovskites. *Scientific Reports* **2016**, *6*, 28618.
